# Supplementary figures and images for: Specific binding of human P[28] rotavirus VP8* protein to blood group ABH antigens on type 1 chains
Source: PLoS Pathog. 2025 Jul 21;21(7):e1013298. doi: 10.1371/journal.ppat.1013298 (PMC12289080; doi:10.1371/journal.ppat.1013298)

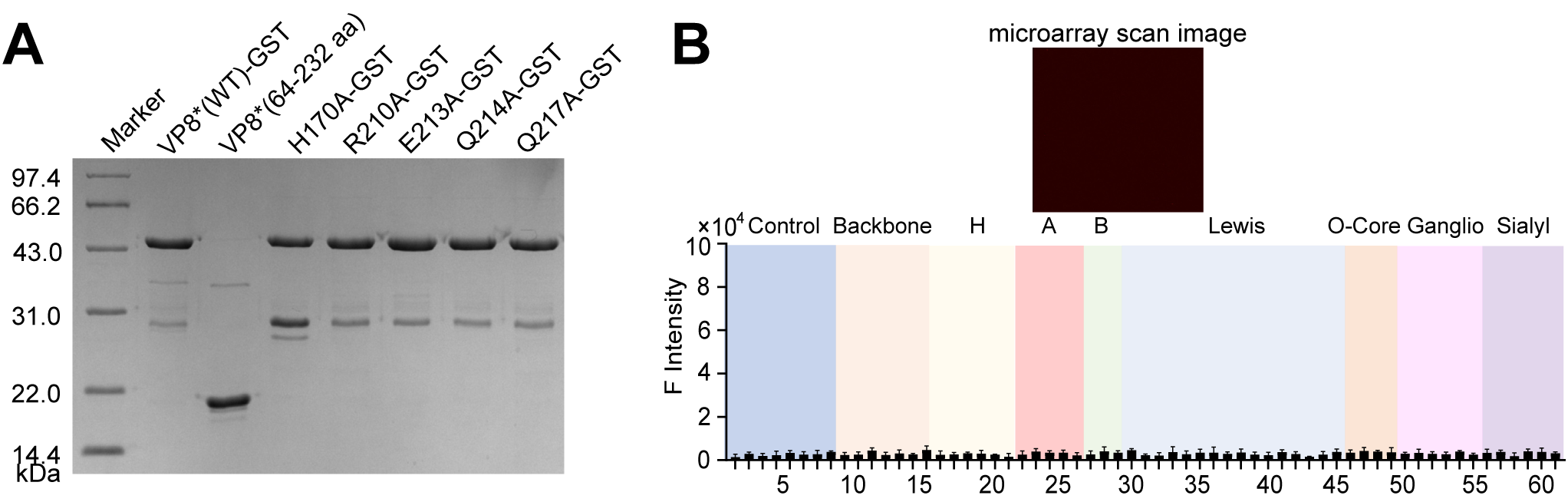

Supplement: S1 Fig — (TIF) [file ppat.1013298.s001.tif]

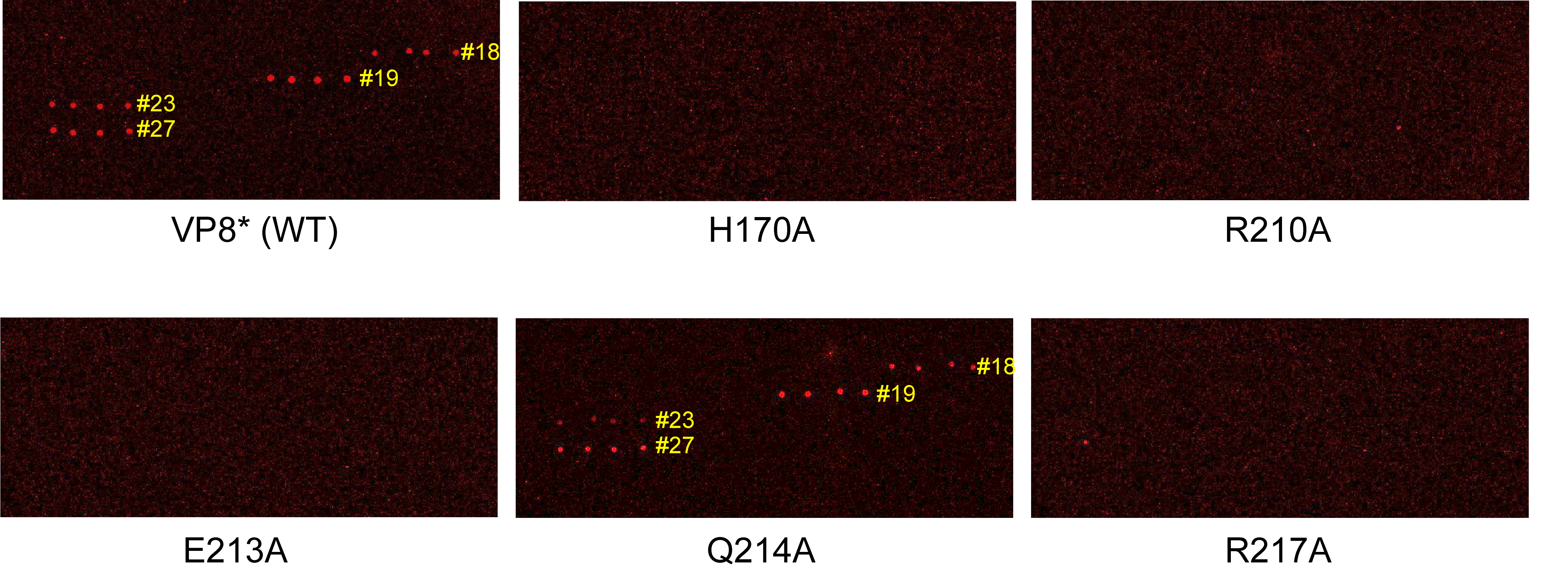

Supplement: S2 Fig — (TIF) [file ppat.1013298.s002.tif]
