# Supplementary material for: Specific binding of human P[28] rotavirus VP8* protein to blood group ABH antigens on type 1 chains
Source: PLoS Pathog. 2025 Jul 21;21(7):e1013298. doi: 10.1371/journal.ppat.1013298 (PMC12289080; doi:10.1371/journal.ppat.1013298)
Supplement: S1 Table — (DOCX) [file ppat.1013298.s003.docx]

**S1 Table.** List of glycan probes in microarrays.

| **No.** | **Probe name** | **Sequences** |
| --- | --- | --- |
| 1 | PBS | — |
| 2 | BABI | — |
| 3 | Thr-BABI | Threonine-BABI |
| 4 | Lac-BABI | Galβ1-4Glc-BABI |
| 5 | M6-BABI | Manβ1-4GlcNAcβ1-4GlcNAc-BABI  2Manα1  Manα1-2Manα1  Manα1  6  -  3  -  6  -  3  -  Manα1 |
| 6 | M7-BABI | Manβ1-4GlcNAcβ1-4GlcNAc-BABI  Manα1-2Manα1-2Manα1  Manα1  Manα1  6  -  3  -  6  -  3  -  Manα1 |
| 7 | M8-BABI | Manβ1-4GlcNAcβ1-4GlcNAc-BABI  Manα1-2Manα1  Manα1-2Manα1  Manα1-2Manα1  6  -  3  -  6  -  3  -  Manα1 |
| 8 | M9-BABI | Manβ1-4GlcNAcβ1-4GlcNAc-BABI  Manα1-2Manα1-2Manα1  Manα1-2Manα1  Manα1-2Manα1  6  -  3  -  6  -  3  -  Manα1 |
| 9 | Forssman-BABI | GalNAcα1-3GalNAcβ1-3Galα1-4Galβ1-4Glc-BABI |
| 10 | LNT-BABI | Galβ1-3GlcNAcβ1-3Galβ1-4Glc-BABI |
| 11 | pLNH-BABI | Galβ1-3GlcNAcβ1-3Galβ1-4GlcNAcβ1-3Galβ1-4Glc-BABI |
| 12 | LNnT-BABI | Galβ1-4GlcNAcβ1-3Galβ1-4Glc-BABI |
| 13 | LNnH-BABI | Galβ1-4Glc-BABI  Galβ1-4GlcNAcβ1  4  -  Galβ1-4GlcNAcβ1  -  3 |
| 14 | pLNnH-BABI | Galβ1-4GlcNAcβ1-3Galβ1-4GlcNAcβ1-3Galβ1-4Glc-BABI |
| 15 | LnNO-BABI | Galβ1-4GlcNAcβ1-3Galβ1-4GlcNAcβ1-3Galβ1-4GlcNAcβ1-3Galβ1-4Glc-BABI |
| 16 | 2’FL-BABI | Fucα1-2Galβ1-4Glc-BABI |
| 17 | DFL-BABI | Glc-BABI  Fucα1-2Galβ1  -  4  3  -  Fucα1 |
| 18 | H-T1-Penta-BABI | Fucα1-2Galβ1-3GlcNAcβ1-3Galβ1-4Glc-BABI |
| 19 | H-T1-Deca-BABI | Galβ1-4Glc-BABI  Fucα1-2Galβ1-3GlcNAcβ1-3Galβ1-4GlcNAcβ1  6  -  Fucα1-2Galβ1-3GlcNAcβ1  -  3 |
| 20 | Globo H-BABI | Fucα1-2Galβ1-3GlcNAcβ1-3Galα1-4Galβ1-4Glc-BABI |
| 21 | H-T2-Penta-BABI | Fucα1-2Galβ1-4GlcNAcβ1-3Galβ1-4Glc-BABI |
| 22 | A-T1-Tetra-BABI | GalNAcβ1-3Galβ1-3GlcNAc-BABI  Fucα1  -  2 |
| 23 | A-T1-Hexa-BABI | Fucα1  2  GalNAcβ1-3Galβ1-3GlcNAcβ1-4Galβ1-4Glc-BABI  - |
| 24 | Globo A-BABI | GalNAcα1-3Galβ1-3GlcNAcβ1-3Galα1-4Galβ1-4Glc-BABI  Fucα1  2  - |
| 25 | A-T2-Tetra-BABI | GalNAcα1-3Galβ1-4GlcNAc-BABI  Fucα1  2  - |
| 26 | A-T2-Hexa-BABI | Fucα1  2  -  GalNAcα1-3Galβ1-4GlcNAcβ1-4Galβ1-4Glc-BABI |
| 27 | B-T1-Hexa-BABI | Fucα1  2  -  Galα1-3Galβ1-3GlcNAcβ1-4Galβ1-4Glc-BABI |
| 28 | Globo B-BABI | Galα1-3Galβ1-3GlcNAcβ1-3Galα1-4Galβ1-4Glc-BABI  Fucα1  2  - |
| 29 | B-T2-Hexa-BABI | Fucα1  2  -  Galα1-3Galβ1-4GlcNAcβ1-4Galβ1-4Glc-BABI |
| 30 | 3FL-BABI | Glc-BABI  Galβ1  -  4  3  -  Fucα1 |
| 31 | Le^a^-Penta-BABI | GlcNAcβ1-3Galβ1-4Glc-BABI  Fucα1  -  4  Galβ1  3  - |
| 32 | Le^a^-Hexa-BABI | Glc-BABI  GlcNAcβ1-3Galβ1  Fucα1  -  4  4  -  -  3  Fucα1  Galβ1  -  3 |
| 33 | Le^a&x^-Nona-BABI | Galβ1-4Glc-BABI  GlcNAcβ1  6  -  GlcNAcβ1-3Galβ1  Fucα1  -  4  4  -  Galβ1  -  3  -  3  Fucα1  Galβ1  -  3 |
| 34 | Le^a&x^-Octo-BABI | Galβ1-4Glc-BABI  GlcNAcβ1  6  -  Galβ1  Fucα1  4  -  GlcNAcβ1  3  -  Galβ1  3  -  Fucα1  4  -  3  - |
| 35 | Le^a&x^-Deca-BABI | Galβ1-4 GlcNAcβ1-3Galβ1-4Glc-BABI  GlcNAcβ1  6  -  Galβ1  Fucα1  4  -  GlcNAcβ1  3  -  Galβ1  3  -  Fucα1  4  -  3  - |
| 36 | Le^x^-Penta-BABI | GlcNAcβ1-3Galβ1-4Glc-BABI  Galβ1  -  4  Fucα1  3  - |
| 37 | Le^x^-Hexa-BABI | GlcNAcβ1-3Galβ1-4Glc-BABI  Galβ1  -  4  Fucα1  2  -  Fucα1  3  - |
| 38 | Le^x^-Deca-BABI | Galβ1-4GlcNAcβ1-3Galβ1-4Glc-BABI  GlcNAcβ1  6  -  GlcNAcβ1  Fucα1  -  4  4  -  Galβ1  -  3  -  3  Fucα1  Galβ1  -  3 |
| 39 | H&Le^x^-Octo-BABI | 6  -  Fucα1-2Galβ1-3GlcNAcβ1  GlcNAcβ1  Galβ1  Galβ1-4Glc-BABI  3  -  Fucα1  4  -  3  - |
| 40 | H&Le^x^-Deca-BABI | 6  -  Galβ1-3GlcNAcβ1  GlcNAcβ1-3Galβ1-4GlcNAcβ1  Galβ1-4Glc-BABI  3  -  Fucα1  Fucα1-2Galβ1  4  -  3  - |
| 41 | Le^b^-Hexa-BABI | Fucα1  GlcNAcβ1-3Galβ1-4Glc-BABI  Fucα1-2Galβ1  4  -  3  - |
| 42 | Le^b^-Deca1-BABI | Fucα1  Galβ1-4Glc-BABI  GlcNAcβ1-3Galβ1-4GlcNAcβ1  Galβ1-3GlcNAcβ1  6  -  3  -  Fucα1-2Galβ1  4  -  3  - |
| 43 | Le^b^-Octo-BABI | Fucα1  Galβ1-4Glc-BABI  Galβ1-4GlcNAcβ1  GlcNAcβ1  6  -  3  -  Fucα1-2Galβ1  4  -  3  - |
| 44 | Le^b^-Deca2-BABI | Fucα1  Galβ1-4GlcNAcβ1-3Galβ1-4Glc-BABI  Galβ1-4GlcNAcβ1  GlcNAcβ1  6  -  3  -  Fucα1-2Galβ1  4  -  3  - |
| 45 | Le^y^-Hexa-BABI | GlcNAcβ1-3Galβ1-4Glc-BABI  Fucα1-2Galβ1  4  -  3  -  Fucα1 |
| 46 | Core 1-Thr-BABI | Galβ1-3GalNAcα1-Threonine-BABI |
| 47 | Core 2-Thr-BABI | Galβ1  GlcNAcβ1  GalNAcα1-Threonine-BABI  6  -  3  - |
| 48 | Core 3-Thr-BABI | GlcNAcβ1-3GalNAcα1-Threonine-BABI |
| 49 | Core 4-Thr-BABI | GlcNAcβ1  GlcNAcβ1  GalNAcα1-Threonine-BABI  6  -  3  - |
| 50 | aGM-BABI | Galβ1-3GalNAcβ1-4Galβ1-4Glc-BABI |
| 51 | GM1a-BABI | Galβ1-4Glc-BABI  Galβ1-3GalNAcβ1  Neu5Acα2  4  -  3  - |
| 52 | GM1b-BABI | Neu5Acα2-3Galβ1-3GalNAcβ1-4Galβ1-4Glc-BABI |
| 53 | GD1a-BABI | Galβ1-4Glc-BABI  Neu5Acα2-3Galβ1-3GalNAcβ1  Neu5Acα2  4  -  3  - |
| 54 | GD1b-BABI | Galβ1-4Glc-BABI  Galβ1-3GalNAcβ1  Neu5Acα2-8Neu5Acα2  4  -  3  - |
| 55 | GT1a-BABI | Galβ1-4Glc-BABI  Neu5Acα2-8Neu5Acα2-3Galβ1-3GalNAcβ1  Neu5Acα2  4  -  3  - |
| 56 | 3’SL-BABI | NeuAcα2-3Galβ1-4Glc-BABI |
| 57 | 6’SL-BABI | NeuAcα2-6Galβ1-4Glc-BABI |
| 58 | LSTa-BABI | NeuAcα2-3Galβ1-3GlcNAcβ1-3Galβ1-4Glc-BABI |
| 59 | LSTb-BABI | 6  -  3  -  GlcNAcβ1-3Galβ1-4Glc-BABI  NeuAcα2  Galβ1 |
| 60 | LSTc-BABI | NeuAcα2-6Galβ1-3GlcNAcβ1-3Galβ1-4Glc-BABI |
| 61 | DSLNT-BABI | 6  -  3  -  GlcNAcβ1-3Galβ1-4Glc-BABI  NeuAcα2  NeuAcα2-3Galβ1 |

Man: mannose; Fuc: fucose; Gal: galactose; Glc: glucose; NAc: N-acetyl, Neu5Ac: N-acetylneuraminic acid; BABI: the covalent probe linker, 2-(2-(bis(2-aminoethyl)amino)ethyl)-6-hydroxy-1H-benzo[de]isoquinoline-1,3(2H)-dione.
