# Supplementary material for: Specific binding of human P[28] rotavirus VP8* protein to blood group ABH antigens on type 1 chains
Source: PLoS Pathog. 2025 Jul 21;21(7):e1013298. doi: 10.1371/journal.ppat.1013298 (PMC12289080; doi:10.1371/journal.ppat.1013298)
Supplement: Attachment — Submitted filename: Response to Reviewers.docx [file ppat.1013298.s005.docx]

**Reviewers' Comments:**

**Part I – Summary**

**Reviewer #1:** The article presented by Zheng and collaborators describes the interaction of a human rotavirus VP8* belonging to the P[28] genotype with specific histo-blood group antigens (HBGAs). The interaction of human rotaviruses with glycans, such as the HBGAs, has win relevance in the last years due to its implication in host species restriction and genetic susceptibility to infections in humans amongst others.

The authors have focused their attention to a rare human genotype P[28] that is closely related to bat rotaviruses and link the putative cross-species jump to the different ability of this genotype to bind most relevant human HBGAs as they are the H antigen, the A blood group and the B blood group.

The reviewer believes that the methodology used is appropriated to obtain the presented results and that the conclusions are aligned with the results obtained. Several technologies were successfully applied including glycan microarrays, crystallography, biolayer interferometry and site directed mutagenesis all of them confirming the results presented by the authors.

We appreciate the reviewer’s positive comments and the overall approval.

**Reviewer #2**: The biochemical data seems solid. However, there is no virological data to validate the findings. Without it, it is hard to interpret how significant the results are. For example, the VP4 mutations outlined in Fig. 4D cannot be validated without functional analysis.

We are pleased that this reviewer considers our biochemical data solid. We also appreciate and understand the important point raised. However, despite every effort made during revision, regrettably we have been unsuccessful to obtain the desired results, due mainly to the unavailability of the virus strain and the major challenge in reverse genetics of the P genotypes, as explained in the detailed response below.

**Reviewer #3**: This study investigated the glycan-binding specificity of the rotavirus P[28] VP8 protein with respect to type 1 chain ABH antigens, elucidating its unique interaction mechanism. By integrating X-ray crystallography, glycan microarray analysis using a dedicated probe library, bio-layer interferometry, site-specific mutagenesis, and molecular docking and dynamics simulations, it was revealed that the P[28] VP8 exhibits broad HBGA binding, which is distinct from the prevalent human rotavirus strains P[8], P[4], and P[6]. These findings provide experimental evidence for interspecies strain reassortment within the P[I] genogroup and highlight the need for enhanced surveillance to address potential cross-species transmission risks associated with P[I] rotaviruses.

We greatly appreciate the reviewer’s the positive comments and overall approval of our work.

**Part II – Major Issues: Key Experiments Required for Acceptance**

**Reviewer #1**:

This reviewer has not found major issues in this article

We are pleased to have this reviewer’s overall approval of our study.

**Reviewer #2**:

While the genetic manipulation of host cells to remove specific glycans would be challenging, with the current reverse genetics system, the authors should be able to rescue recombinant rotaviruses (monoreassortant viruses in SA11 background for instance) carrying these mutations and test how the infectivity is affected.

We appreciate very much the very valuable point raised by the reviewer. It would be important and ideal to rescue recombinant rotaviruses with engineered mutations to evaluate their impact on viral infectivity. Employing reverse genetics techniques to generate recombinant rotaviruses for validation would indeed strengthen our work considerably. In fact, this was in our plan prior to submission.

In our laboratory, we had already successfully established reverse genetic platforms utilizing the LLR and SA11 strains as backbone vectors. Leveraging these platforms, we accomplished the insertion of VP7-encoding genes and subsequent virus rescue for multiple G genotypes (including G1, G2, G3, G4, G8, and G9). However, significant challenges emerged when attempting to insert VP4-encoding genes for the rescue of P genotype rotaviruses (J Med Virol, 2024, 96, 70065). In response to the suggestions from both this reviewer and **Reviewer 3**, we dedicated substantial time and resources to optimizing the reverse genetics protocol specifically for the P[28] genotype; regrettably, these efforts did not yield successful virus rescue.

These findings align with historical (e.g. Greenberg et al., J. Gen. Virol. 1983, 64, 313–320; Midthun et al., J. Virol. 1985, 53, 949–954) and recent work (e.g. Kawagishi, et al., J. Virol. 2020, 94. e00963-19 and Falkenhagen et al., Viruses, 2020, 12, 201) from other laboratories reporting difficulties and challenges in VP4-based virus rescue. For instance, Falkenhagen and colleagues successfully rescued G2, G9, and G12 genotypes using the SA11 backbone; yet, when attempting to insert P[4], P[6], and P[8] VP4-encoding genes, they failed P[4] and P[8], while P[6] requiring four consecutive blind passages after transfection to exhibit detectable cytopathic effects.

Collectively, these results point to a substantially greater technical hurdle during reverse genetics-based virus rescue for the VP4 genotypes compared to the G genotypes. With respect, while we recognize the importance of further exploration in this area, we believe that resolving the challenges associated with P genotype rotavirus rescue via reverse genetics may extend beyond the scope of the present study and represents a distinct research direction that warrants a separate investigation.

We have now pointed out this limitation of our present study in the Discussion (Page 19 Lines 333-336; revised manuscript with marked changes).

**Reviewer #3**:

1. This study primarily investigates the P[28] genotype, need to augment data on the prevalence of P[28] genotype viruses among human rotaviruses to underscore its epidemiological significance.

We appreciate the reviewer's suggestion but feel sorry for the confusion caused. Currently, P[28] genotype has only been detected in Ecuador and Suriname, South America, and is not a widely prevalent genotype. However, based on the recent work by Esona and colleagues (Ref 23), the genetically close relationship of P[28] strain to a bat strain suggest that G20P[28] is of bat origin. Bats have been recognized as an important natural reservoir of zoonotic viruses of different families and this may have future epidemiological significance.

We have now modified the description in Introduction (Page 5 Line 89; revised manuscript with marked changes) and Discussion (Page 19 Lines 330-331) to clarify this point.

2. The study's conclusions are largely based on the P[28]-VP8* recombinant protein. Although the in vitro experiments are thorough and detailed, they lack validation at the cellular level. For instance, assessing the change in infection titer after pre-incubating glycans such as H-T1-Penta with the P[28] genotype rotavirus before infecting cells is essential, it is necessary to demonstrate whether HBGA is a key receptor in the infection process of P[28] rotavirus.

We agree with the reviewer that demonstration, at the cellular level, whether HBGA is a key receptor in the infection process of P[28] rotavirus is very important. As mentioned above, P[28] genotype is currently not a circulating strain, and has only been detected in Ecuador and Suriname, South America. We do not currently have this strain in our laboratory or any laboratories in China. We have tried many different ways to contact the Esona group at the US CDC (Ref 23), and Gabriel Trueba (Universidad San Francisco de Quito, Quito, Ecuador) and Joseph N. S. Eisenberg (University of Michigan, Ann Arbor) without success. It may be understandable that under the present climate shipment of virus to China is not feasible. Unfortunately, the unavailability of P[28] rotavirus prevented us to conduct the infection experiment.

3. Figure 2B evaluated four glycans (H-T1-Penta, H-T1-Deca, A-T1-Hexa, and B-T1-Hexa); however, only three were further analyzed for their interactions with VP8* in Figure 2C and subsequent experiments. This discrepancy requires clarification to ensure completeness of the dataset.

The reviewer is right that only three glycans (A-T1, B-T1 and H-T1) were used for BLI analysis (**Fig 2C**) while four (including an additional H-T1 glycan: the H-T1 decasaccharide) were used for inhibition analysis (**Fig 2B**). Inhibition of binding was carried out on arrays and therefore the usage of materials is limited. However, a large amount of material is required for BLI. We have reasonable stock of the monovalent HBGA H-T1, A-T1 and B-T1 penta- and hexasaccharides, but the amount of the more complex bivalent H-T1-decasaccharide, with two H epitopes on each of the two branches, is limited in amount as its isolation from human milk is difficult. The microarray experiments already indicated a stronger binding with the bivalent than the monovalent H-T1. Therefore, for the two reasons further BLI experiment H-T1 decasaccharide was not used.

4. Figure 4D confirmed four key amino acid sites. If the rotavirus with site mutations can be rescued using a reverse genetics system and the effects of these mutations on the virus's infectivity and binding activity with various glycans are further analyzed, the conclusions of this study might be strengthened.

We thank **Reviewer 3** for this important point, which was similarly raised by **Reviewer 2**.

We agree entirely with both reviewers in that recombinant mutations constructed by reverse genetics techniques for validation can indeed significantly strengthen our study. As we explained to **Reviewer 2**, in our laboratory, we have already successfully established reverse genetic platforms utilizing the LLR and SA11 strains as backbone vectors. Leveraging these platforms, we accomplished the insertion of VP7-encoding genes and subsequent virus rescue for multiple G genotypes (including G1, G2, G3, G4, G8, and G9). However, significant challenges emerged when attempting to insert VP4-encoding genes for the rescue of P genotype rotaviruses (J Med Virol, 2024, 96, 70065). In response to the suggestions from both this reviewer and **Reviewer 2**, we dedicated substantial time and resources to optimizing the reverse genetics protocol specifically for the P[28] genotype; regrettably, these efforts did not yield successful virus rescue.

These findings align with historical (e.g. Greenberg et al., J. Gen. Virol. 1983, 64, 313–320; Midthun et al., J. Virol. 1985, 53, 949–954) and recent work (e.g. Kawagishi, et al., J. Virol. 2020, 94. e00963-19 and Falkenhagen et al., Viruses, 2020, 12, 201) from other laboratories reporting difficulties and challenges in VP4-based virus rescue. For instance, Falkenhagen and colleagues successfully rescued G2, G9, and G12 genotypes using the SA11 backbone; yet, when attempting to insert P[4], P[6], and P[8] VP4-encoding genes, they failed P[4] and P[8], while P[6] requiring four consecutive blind passages after transfection to exhibit detectable cytopathic effects.

Collectively, these results point to a substantially greater technical hurdle during reverse genetics-based virus rescue for the VP4 genotypes compared to the G genotypes. With respect, while we recognize the importance of further exploration in this area, we believe that resolving the challenges associated with P genotype rotavirus rescue via reverse genetics may extend beyond the scope of the present study and represent a distinct research direction that warrants a separate investigation.

We have now pointed out this limitation of our present study in the Discussion (Page 19 Lines 333-336).

**Part III – Minor Issues: Editorial and Data Presentation Modifications**

**Reviewer #1**:

Although the reviewer does not appreciate any major concerns there are some minor issues that should be improved:

Line 66. The authors state that the human P[11] genotype recognizes both type I and type II precursors, but the articles cited show that this genotype binds type II precursors (Galb1-4GlcNAc) but not type I (Galb1-3GlcNAc).

We have realized the mistake and apologize for the incorrect citation. We have now inserted the correct reference (new Ref 12: Hu et al., Nat Commun. 2015, 6, 8346), which describes human P[11]-VP8* recognition to both type I and type II precursors.

Line 68. The authors state that there are at least 38 P genotypes in group A rotavirus, but this number has increased dramatically since 2020, currently there are at least 58 P genotypes (https://rega.kuleuven.be/cev/viralmetagenomics/virus-classification/rcwg). Please update the number and refer to the website that keeps the rotavirus genotype updated.

We appreciate the reviewer’s suggestion and have now revised the description accordingly (Page 4 Lines 68-69; revised manuscript with marked changes).

Line 136. The authors found that, contrary to other human rotaviruses, the P[28] VP8* utilized in this study was not able to bind the precursors. This fact should be further discussed in the discussion section. Please notice that for instance in the P[8] genotype interactions with HBGAs the fucose has not direct interactions with the protein (Gozalbo-Rovira et al., 2019) while the results presented here show direct interactions of the fucose with the protein.

We appreciate very much the point raised and feel sorry for not having described our results clearly.

Indeed, the negative binding signals exhibited from type 1 and type 2 backbone chains in our microarray binding analysis may be different from several other prior studies. In the co-crystal structure of P[28]-VP8*-LNFP I complex, we observed that the fucose-C6 in LNFP I is located within 4.0 Å of the R210 side chain, forming a hydrophobic interaction with R210, consistent with the similar result of P[6]-VP8* interaction with LNFP I (3.9 Å, Nat Commun. 2018, 9, 2631). We have now modified the description of the results to clearly indicate this interaction (Page 10 Lines 185-188). As reported previously (e.g. PLoS Pathog, 2019, 15, e1007865), even without direct interaction with the protein, fucose may still play an important role, e.g. altering the conformation of LNFP I and thereby increasing its binding affinity to VP8*.

As suggested, we have now discussed this in the Discussion section (Page 18 Lines 304-308).

Line 145. In this paragraph the authors describe their results obtained by biolayer interferometry but it is not clear here, nor in the material and methods section (line 373), the source and the nature of the oligosacchares used, please provide that information.

We are sorry for the missing information. We have now inserted the source of these materials (Elicityl, Grenoble, France) in the Materials and Methods section.

Line 154. In the text to figure 2B it is not clear which sugar was used to block the binding in each case. Please improve the description of this panel.

With respect, we feel that the description of the glycans used for inhibition is adequate: the binding to H-T1-Penta and H-T1-Deca probes were inhibited by “LNFP I”, and similarly, the inhibitory effects can be found for the A-T1-Hexa and B-T1-Hexa probes by “respective hexasaccharide” (ie by A-T1 and B-T1 hexasaccharide, respectively).

Line 281. There are mistakes in the T1 and T2 descriptions T1 should be Galb1-3GlcNac and T2 Galb1-4GlcNac. Please correct

We are grateful to this reviewer for pointing out the mistake and we have now made the correction in the revised manuscript (Page 17 Line 295).

**Reviewer #2**:

Statistics are missing from all the panels (Fig. 2B, 4A, and 4D).

As pointed out by the reviewer, we have now added statistical analysis in these figures.

**Reviewer #3:**

1. In the background section, the authors propose that the P[28] strain may have originated from reassortment between human and bat rotaviruses. Further explanation is needed on which genes may have experienced reassortment. Given this study's focus on the VP8* segment, the potential reassortment of the VP4 fragment is pertinent to the research's significance.

We apologize for the confusion caused. The reassortment was actually considered by Esona and colleagues (Ref 23) following their extensive molecular characterization of a human G20P[28] rotavirus A strain with multiple genes related to bat rotaviruses. The found that the VP7, VP4, VP6, NSP3 and NSP5 genes of the strain RVA/Human-wt/SUR/2014735512/2013/G20P[28] are nearly identical to the bat strain 3081/BRA and bat-like human strain Ecu534. This suggests that these genes, and possibly the strain itself, are of bat origin, as they found that the VP7, NSP3 and NSP5 gene segments of P[28] Suriname strain and the VP7 gene segments of P[28] Ecuadorian strain are closely related to gene sequences of the bat 3081/BRA strain and may be reassortment between human and bat RVs. Although the VP4s of Suriname strain and Ecuadorian strain share a nucleotide (amino acid) similarity of 84.6% (97.5%), the VP4 gene fragments of them did not show similarities to that of bat RV. Therefore, we did not consider the recombination of VP4.

2. The introduction section needs additional cited references, such as in lines 58-59: "The P genotypes are further classified into five genogroups [1], P[I] to P[V], based on the sequences of the spike protein VP8*, one of the two proteolytic fragments of VP4." And in lines 73-74: "The glycan bindings of animal strains of P[1], P[2], P[3], and P[7] are reported to be sialic acid-dependent."

As suggested, we have now inserted the relevant references (Refs 1&13, and Refs 15-20) at the respective places in the revised manuscript.

3. In line 68, the authors state, "There have been at least 38 P genotypes identified until 2020." However, existing research has reported more than 50 P genotype rotaviruses, so this description needs to be updated.

As pointed out by this reviewer, we have updated this information and quoted its source: https://rega.kuleuven.be/cev/viralmetagenomics/virus-classification/rcwg. (Page 4 Lines 68-69; revised manuscript with marked changes)

4. Please discuss why the structural differences between P[28] and other P[I] types, such as P[5]-VP8, are more pronounced than those in other types, like P[9] and P[14]-VP8 (Figure 1B).

Thanks for the suggestion. The Values represent root mean square deviations (RMSDs, unit: Å) of the Cα atoms of one VP8* monomer. The RMSD values partly reflect the similarity of the structure and do not have absolute relation with the phylogenetic analysis of the sequences. In this study, P[28] VP8*structure displayed closest value to that of P[7] VP8*. According to the previous report, P[7] VP8* structure showed closer values to that of P[9] and P[14], similar to that of P[28] (Front Mol Biosci, 2021, 8, 658029). For the P[5] VP8*, it was found that P[5] VP8* structure was most similar that of P[8] VP8* (Virology, 2021, 563, 116-125.). It is understandable that the structural differences between P[28] and P[5] are more pronounced than those of P[9] and P[14] VP8*s. We have added the discussion in the revised manuscript as suggested (Page 6 Lines 117-123).

5. In Figure S1, each VP8* protein exhibits moderate purity. Notably, the GST-fused VP8 proteins display significant impurity bands between 31-43 kDa. A discussion is warranted to determine whether these impurities might impact the relevant experimental results.

We are sorry for not having been explained these bands. The bands are the free GST tag (~26kDa) as previously reported (e.g. J Virol. 2012, 86, 4833-43; J Virol. 2012, 86, 9899-910; J Virol. 2018, 29, 14). To be certain of this impurity not involved in the interaction, we have carried out an additional microarray binding experiment using GST protein alone, demonstrating that the free GST protein does not influence the relevant experimental results. The result has now been inserted as **S1 Fig B**.

6. Please discuss the limitations of the article.

As required, we have inserted discussions on the limitations of our work in the revised manuscript (Page 19 Lines 330-336), including i) lack of epidemiological analysis data as P[28] is not a prevalent genotype with limited case report, and ii) the work is largely based on the P[28]-VP8* recombinant protein, lacking validation at the cellular level of virus infection.

7. In the last sentence of the discussion section, it is mentioned that the monitoring of the P[28] strain should not be neglected. Is this statement too absolute? Although the monitoring of the P[28] genotype has certain epidemiological value, more data on circulating viruses is still needed to support it.

We agree with the reviewer. As this is only speculative, we have toned down the statement (Page 19 Lines 339-340).
